# Reversible Snapping of Constrained Anisotropic Hydrogels Upon Light Stimulations

**DOI:** 10.1002/advs.202402824

**Published:** 2024-05-05

**Authors:** Chen Fei Dai, Qing Li Zhu, Olena Khoruzhenko, Michael Thelen, Huiying Bai, Josef Breu, Miao Du, Qiang Zheng, Zi Liang Wu

**Affiliations:** ^1^ Ministry of Education Key Laboratory of Macromolecular Synthesis and Functionalization Department of Polymer Science and Engineering Zhejiang University Hangzhou 310058 China; ^2^ Bavarian Polymer Institute and Department of Chemistry University of Bayreuth Universitätsstrasse 30 95440 Bayreuth Germany

**Keywords:** anisotropic hydrogels, fast actuation, nanosheets, snapping, soft actuators

## Abstract

Creatures, such as Venus flytrap and hummingbirds, capable of rapid predation through snap‐through transition, provide paradigms for the design of soft actuators and robots with fast actions. However, these artificial “snappers” usually need contact stimulations to trigger the flipping. Reported here is a constrained anisotropic poly(*N*‐isopropylacrylamide) hydrogel showing fast snapping upon light stimulation. This hydrogel is prepared by flow‐induced orientation of nanosheets (NSs) within a rectangular tube. The precursor containing gold nanoparticles is immediately exposed to UV light for photopolymerization to fix the ordered structure of NSs. Two ends of the slender gel are clamped to form a buckle with bistability nature, which snaps to the other side upon laser irradiation. Systematic experiments are conducted to investigate the influences of power intensity and irradiation angle of the laser, as well as thickness and buckle height of the gel, on the snapping behaviors. The fast snapping is further used to kick a plastic bead and control the switch state. Furthermore, synergetic or oscillated snapping of the gel with two buckles of opposite directions is realized by inclined irradiation of a laser or horizontal irradiation with two lasers, respectively. Such light‐steered snapping of hydrogels should merit designing soft robots, energy harvests, etc.

## Graphical abstract

Reversible snap‐through transition is realized in the constrained anisotropic hydrogel strip upon light irradiation, leading to accumulation and sudden release of the elastic energy that produces speedy action and large stroke.

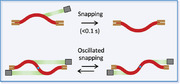

## 1 Introduction

Nature provides numerous inspirations to devise artificial soft actuators and robots.^[^ ^1^ ^]^ By combining the responsiveness of soft materials and elaborate structural designs, various soft actuators and robots have been developed with diverse morphing and motion modes.^[^ ^1^, ^2^ ^]^ Among different morphing‐related geometries, the bistable structure has attracted tremendous attention because it possesses two stable configurations at a constant condition, which can be switched via snap‐through transition triggered by mechanical force or external stimuli.^[^ ^3^ ^]^ For example, hummingbirds undergo a rapid beak closure in a few milliseconds to capture the flying insects by harnessing the snap‐through instability of the bistable structure.^[^ ^3b,e^ ^]^ The snapping process generally comprises the accumulation and then sudden release of elastic energy. Snapping happens when the accumulated energy surpasses an energy barrier; the sudden release of energy speeds up the deformation and amplifies the output force. Therefore, the snap‐through transition has several features, including fast action, large stroke, and no extra energy required to maintain the configuration, which in combination are crucial for soft actuators and robots.^[^ ^3e,g^ ^]^ For example, Gorissen et al. have fabricated a soft inflatable jumper having a spherical elastomer cap, which can snap and jump when pressurized.^[^ ^3k^ ^]^ However, this type of artificial systems lacks responsiveness to external stimuli and external force/pressure is required to trigger the snapping.

To address the aforementioned issue, soft materials with responsive matrices and/or responsive fillers are applied to develop soft actuators or robots with bistable structures.^[^ ^4^ ^]^ For example, Fang et al. designed a hydrogel‐based jumper with a flat plate as the main body and two bistable curved strips as the legs.^[^ ^4a^ ^]^ The inner side of the legs was patterned with micro‐channels and thereby capable of fast solvent absorption and swelling via capillary forces. The swelling mismatch led to snap‐through transition of the leg and thus jumping of the gel system. Recently, we also realized electro‐actuated reversible snapping of a domal polyelectrolyte hydrogel.^[^ ^4b^ ^]^ Under electric field, the mobile ions were redistributed within the polyelectrolyte network, which intended to bend to the other side of the dome yet was hampered by the geometric constrain. The elastic energy was accumulated until a threshold, and then a snap‐through transition occurred to partially release the elastic energy. In these studies, stimulations are imposed on the gels in direct‐contact manners, making remote and local stimulations for the snap‐through transition to be a challenging task. In addition, the hydrogel systems usually require tens of seconds to trigger the snapping and/or several minutes for recovery to the original state because the motions are limited by relatively low molecular diffusion process.

Developing soft actuators with contactless stimulus‐triggered snapping is of great significances. Stimuli such as light and magnetic fields are often used to actuate soft materials in a contactless way.^[^ ^5^ ^]^ However, there are only a few efforts devoted to devising soft actuators capable of snapping upon contactless stimulations. Majidi et al. developed a bistable curved strip made of a ferroelastic composite by embedding ferromagnetic microparticles into the polydimethylsiloxane matrix.^[^ ^5a^ ^]^ The strip underwent a snap‐through transition under the action of a magnetic field. However, the strength of magnetic fields rapidly decreases with the distance, and therefore it is challenging to actuate the system precisely and remotely. In alternative, light enables remote stimulation for the materials with a high spatiotemporal resolution. For instance, White et al. developed an azobenzene‐containing liquid crystalline network (LCN) and realized reversible snapping of a buckled LCN film upon light irradiation.^[^ ^5b^ ^]^ The localized contraction at the light‐exposed surface bent the constrained LCN film and triggered the snap‐through transition. However, the morphing amplitude of the LCN‐based snappers is small, and the relaxation after photo‐isomerization is rather slow, leading to prolonged intervals between sequential snapping. There is no report on photo‐triggered reversible and fast snapping of soft actuators especially based on hydrogel materials. It is highly desired to realize light‐driven snap‐through transition in hydrogels to broaden the applications in biomedical devices, soft actuators/robots, etc. The challenge lies in the slow response that cannot immediately generate sufficiently‐high internal stress derived from light stimulation.

Herein, we demonstrate a bistable hydrogel that exhibits a light‐actuated snap‐through transition with a high action speed and a large morphing amplitude. The anisotropic poly(*N*‐isopropylacrylamide) (PNIPAm) hydrogel is prepared by shear‐induced orientation of fluorohectorite nanosheets (NSs) followed by photopolymerization to fix the ordered structure. Gold nanoparticles (AuNPs) are incorporated into the gel to afford response to light. Upon light irradiation, the gel bends toward the light due to photothermal gradient and asymmetric contraction in the thickness direction. A slender gel is constrained to form a buckle, which is characterized by a bistability nature. Under light irradiation on the top of the buckle, the gel tends to bend to the opposite direction yet is hampered by the geometric constrain. The elastic energy is thereby accumulated, leading to a snap‐through transition until the energy surpasses a critical value. Such snapping is reversible and can be repeated for a number of times upon light irradiation from different sides of the gel strip. The snapping behavior can be tuned over a wide range by controlling the gel properties and irradiation conditions. This light‐triggered snapping can be used to execute specific tasks, such as kicking a ball and switching on or off the circuit. We also found collective snapping in a slender gel with two parts buckled to opposite directions. The light‐triggered snapping of one part results in a snap‐through transition of the other without light stimulation, manifesting the synergistic effect. Continuous oscillated snapping is also realized in the gel with two buckles under static irradiation with two parallel light beams. This work should be informative for the design of hydrogel‐based soft actuators and robots with fast actions and large strokes.

## 2 Results and Discussion

### 2.1 Synthesis, Structure, and Response of the Anisotropic Hydrogel

**Figure 1:**
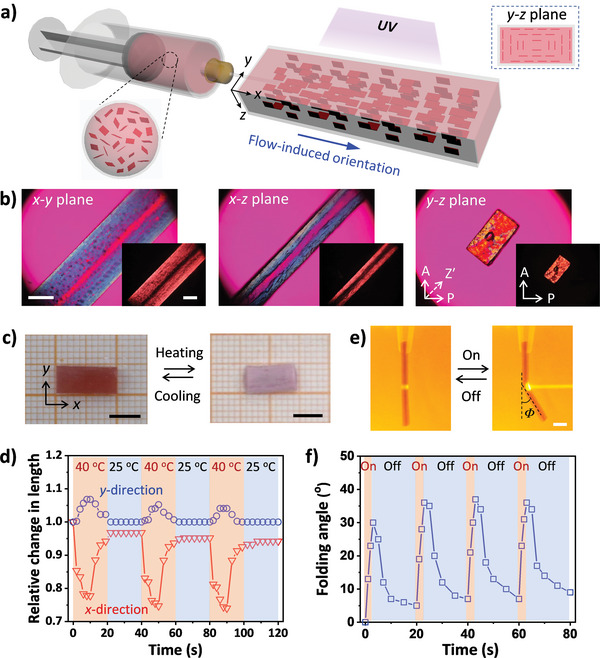
a) Schematic for the synthesis of anisotropic hydrogel by flow‐induced orientation of nanosheets. The ordered structure is fixed by subsequent photopolymerization to form a nanocomposite gel. For simplicity, the monomer, cross‐linker, initiator, and gold nanoparticles are omitted in the scheme. b) Polarized optical microscope images of the gel observed from different directions. A: analyzer, P: polarizer, Z′: slow axis of 530 nm tint plate. c,d) Digital photos (c) and varying dimensions (d) of the gel under cyclic switching the incubation conditions between 25 and 40 °C water baths. e,f) Digital photos (e) and varying folding angle (f) of the gel upon cyclic laser irradiation with the power intensity of 1.98 W cm^−2^. The contents of NSs and AuNPs in the anisotropic gel are 1 and 0.03 wt.%, respectively. The thickness of the gel is 2 mm. Scale bars in (b), (c), and (e) are 2, 5, and 5 mm, respectively.

The synthesis of the anisotropic hydrogel is illustrated in **Figure** **1**a. After injecting the aqueous precursor containing *N*‐isopropylacrylamide (NIPAm), photo‐initiator, cross‐linker, NSs, and AuNPs into a rectangular tube (inner cross‐section dimensions: width of 4 mm, height ranging from 0.5 to 3 mm), the reaction cell is immediately exposed to UV light for photopolymerization. At the NS concentration chosen (1 wt.%), the suspension is in a nematic phase due to the short‐range correlation between NSs with large aspect ratio (≈20 000) and high charge density (1.1 nm^−2^). Long‐range orientation of NSs in the nematic suspension can be achieved by mechanical or flow shear, leading to transition from multidomain to monodomain, which is crucial for the synthesis of anisotropic gel. In this study, the flow shear during the injection of precursor suspension results in homogeneous orientation of the NSs parallel to the surface of the rectangular glass tube. After photopolymerization, the obtained nanocomposite hydrogel has an anisotropic structure, as confirmed by polarizing optical microscope (POM) observations. Without specific notations, the anisotropic gel contains 1 wt.% of NSs and 0.03 wt.% of AuNPs. As shown in Figure 1b, the gel exhibits strong birefringence with a sandwich‐like pattern, when observed from the top and from the side (corresponding to *x‐y* plane and *x‐z* plane, respectively). When observed from the cross‐section (corresponding to *y‐z* plane), the gel exhibits a X‐shaped birefringent pattern. These results indicate that the NSs are aligned along the wall of the tube, forming quasi‐concentric structure with serial rectangular contour lines within the gel strip. Considering the varying shear rates during the injection, the orientation degree of NSs within the gel should gradually decrease from the surface to the central. Such alignments are also verified by small angle X‐ray scattering (SAXS) measurements of the gel (Figure S1, Supporting Information). The 2D scattering patterns in positions near the long side and the short side of the rectangular cross‐section of the gel are similar but orthogonal, indicating the alignment of NSs along the tube wall. From the intensity‐azimuthal plot, the orientation degree of the NSs within the gel is ≈0.8.

The PNIPAm hydrogel strip exhibits an anisotropic response to temperature. When being incubated in 40 °C water bath for 10 s, the gel contracts by 0.78 along the *x* direction and slightly expands by 1.06 along the *y* direction (Figure 1c). The anisotropic response of the gel arises from the increase in electrostatic repulsion between the highly charged NSs, when water molecules are released from the PNIPAm chains at elevated temperature (above the lower critical solution temperature (LCST) of PNIPAm) that results in increased permittivity of the media.^[^ ^6^ ^]^ When the gel is immediately transferred into 25 °C water bath, it recovers original dimensions within 20 s. Such fast and anisotropic deformation of the gel with a short period of action time is almost reversible upon cyclic switching between 25 and 40 °C water baths (Figure 1d). However, after incubation in 40 °C water bath for 2 min, the gel contracts its volume, with shrinkage in length of 0.92 along the *x* direction and 0.69 along the *y* direction (Figure S2a, Supporting Information). The long‐term contraction behavior of the gel is similar to the conventional PNIPAm gel, in which water molecules are released and diffuse out of the matrix. For the anisotropic gel in this study, volume contraction completes after ≈1 h, with shrinkage in length of 0.52 along the *x* direction and 0.65 along the *y* direction (Figure S2b, Supporting Information).

Owing to the presence of AuNPs, the anisotropic gel also shows response to light.^[^ ^7^ ^]^ The absorption spectrum of the gel has a peak at 520 nm, corresponding to the surface plasmon resonance of the AuNPs (Figure S3, Supporting Information), which afford the gel with high photothermal efficiency. The gel can be locally heated upon irradiation of a green laser (wavelength, 520 nm). The rising speed and amplitude of the local temperature depend on the content of AuNPs, as well as the intensity and irradiation time of the laser (Figure S4, Supporting Information). With a higher intensity, the temperature increases rapidly and reaches a saturated temperature. As expected, the temperature increases faster for the gel with a higher content of AuNPs. For example, the temperature of the gel with 0.03 wt.% AuNPs is elevated from 27 to 55 °C within 4 s under laser irradiation with an intensity of 1.98 W cm^−2^. However, it has little influence on the rising speed and amplitude of the local temperature of the gel, when the content of AuNPs is above 0.03 wt.%. Cyclic laser irradiation results in dynamic modulation of the temperature across the LCST of PNIPAm (Figure S5, Supporting Information). Accordingly, the gel folds toward the light due to the photothermal gradient within the gel (Figure 1e).^[^ ^8^ ^]^ With the increase in the irradiation time, the folding angle of the gel increases until it reaches a maximum value of ≈80° (Figure S6, Supporting Information). Reversible folding can be realized by cyclic laser irradiation on the gel strip (Figure 1f; Movie S1, Supporting Information). The fast and reversible response of the anisotropic gel is crucial for the light‐triggered snap‐through transition of a constrained gel strip, which will be described in the following section.

### 2.2 Light‐Actuated Snapping of Buckled Anisotropic Gel

**Figure 2:**
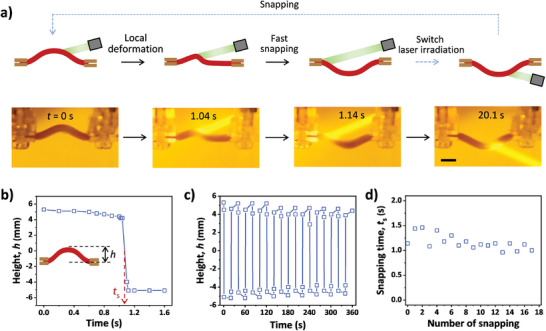
a) Schematic and snapshots to show the reversible snap‐through transition of the buckled hydrogel upon laser irradiation. The elapsed time is shown in the photos. Length and thickness of the slender gel are 20 and 2 mm, respectively. Power intensity, 1.98 W cm^−2^. Scale bar, 5 mm. b) Height variation of the buckled gel as a function of the irradiation time. Snapping time is determined when the height (*h*) changes instantaneously. c,d) Variations of buckle height of the gel upon sequential light irradiation with switched direction (c) and corresponding changes of the snapping time (d).

To realize a snap‐through transition of the gel, the structure should have bistability nature. A simple way to form a buckle is to clamp the two ends of the slender gel with a distance of the two clamps shorter than the length of the gel strip. The constrained gel has bistability nature. When the gel is compressed with a mechanical force from the top of the buckle, bending to the opposite side is hampered by the geometric constrain; after surpass an energy barrier, the gel buckle quickly moves to the other side with the force‐induced snap‐through transition (Figure S7, Supporting Information). In the force‐displacement curve, the initial increase in force suggests energy accumulation in the gel system, and subsequently rapid decrease in force indicates the snap‐through transition. Similar snap transition can be actuated by light. When the gel strip is buckled upward and irradiated from the top by a laser, the light‐irradiated region readily contracts its volume, driving the gel strip to bend downward (**Figure** **2**a). However, this bending is hampered by the geometric constrain, different from the deformation of a gel strip in a free state, as showed in Figure 1e. Consequently, the elastic energy is accumulated in the constrained gel strip until it is above a critical value. In this line, after irradiation for ≈1.1 s, the buckled gel undergoes a sudden snap‐through transition and quickly reaches the other stable configuration to partially release the stored elastic energy (Figure 2a). Similar to reported phenomena,^[^ ^3^, ^9^ ^]^ snapping is easier to happen in this study when the irradiation is slightly off the center, because the initial asymmetric deformation pushes the buckle away from the irradiation that reduces the energy barrier of snapping. However, no snapping occurs if the irradiation has a large offset from the center. Further experiments indicate that the best condition for snapping is the light irradiation on the gel with ≈2.5 mm offset of the center of the buckle. We should note that both oriented NSs and randomly dispersed AuNPs are indispensable for the rapid response and snap‐through transition upon light irradiation. The gels without AuNPs, regardless of having NSs or not, have no response to light (Figure S8, Supporting Information). The gel with AuNPs yet without NSs deforms slightly and cannot snap to the other side under light irradiation, probably because of the slow response and insufficient driving force derived from light stimulation.

The snapping process is analyzed in detail from the recorded movie (Movie S2, Supporting Information). In the first 1.0 s, the irradiated region contracts to some extent, resulting in reduced height of the buckle and accumulation of elastic energy in the constrained gel. A fast snap‐through transition occurs at ≈1.1 s. Specifically, one concavity appears and rapidly grows at the irradiated region, which breaks the structural symmetry and results in snapping that completes within 0.1 s. The snapping is also manifested in the varying height of the buckle as a function of the irradiation time (Figure 2b). The height of the buckle is gradually decreased from to 5.3 to 4.2 mm under laser irradiation before the snapping to be triggered. Subsequently, snapping occurs and leads to a sudden and drastic drop in the height of the buckle from 4.2 to −4 mm. The negative height indicates the buckle is switched to the opposite side with a similar buckle amplitude. The moment with the fastest change of the height is defined as the snapping time, *t*_s_, i.e., the irradiating time required for the snap‐through transition. Considering the structural symmetry of the gel strip, the snapping of the buckled gel is fully reversible and capable of being continuously switched for at least tens of times (Figure 2c). The interval time is set as ≈20 s between sequential light irradiation from opposite sides to guarantee the recovery of local temperature and dimensions of the gel. We should note that the snapping time slightly decreases in successive actuations (Figure 2d), probably due to the incomplete recovery of the gel within an insufficient interval before the next cycle of light stimulation.

**Figure 3:**
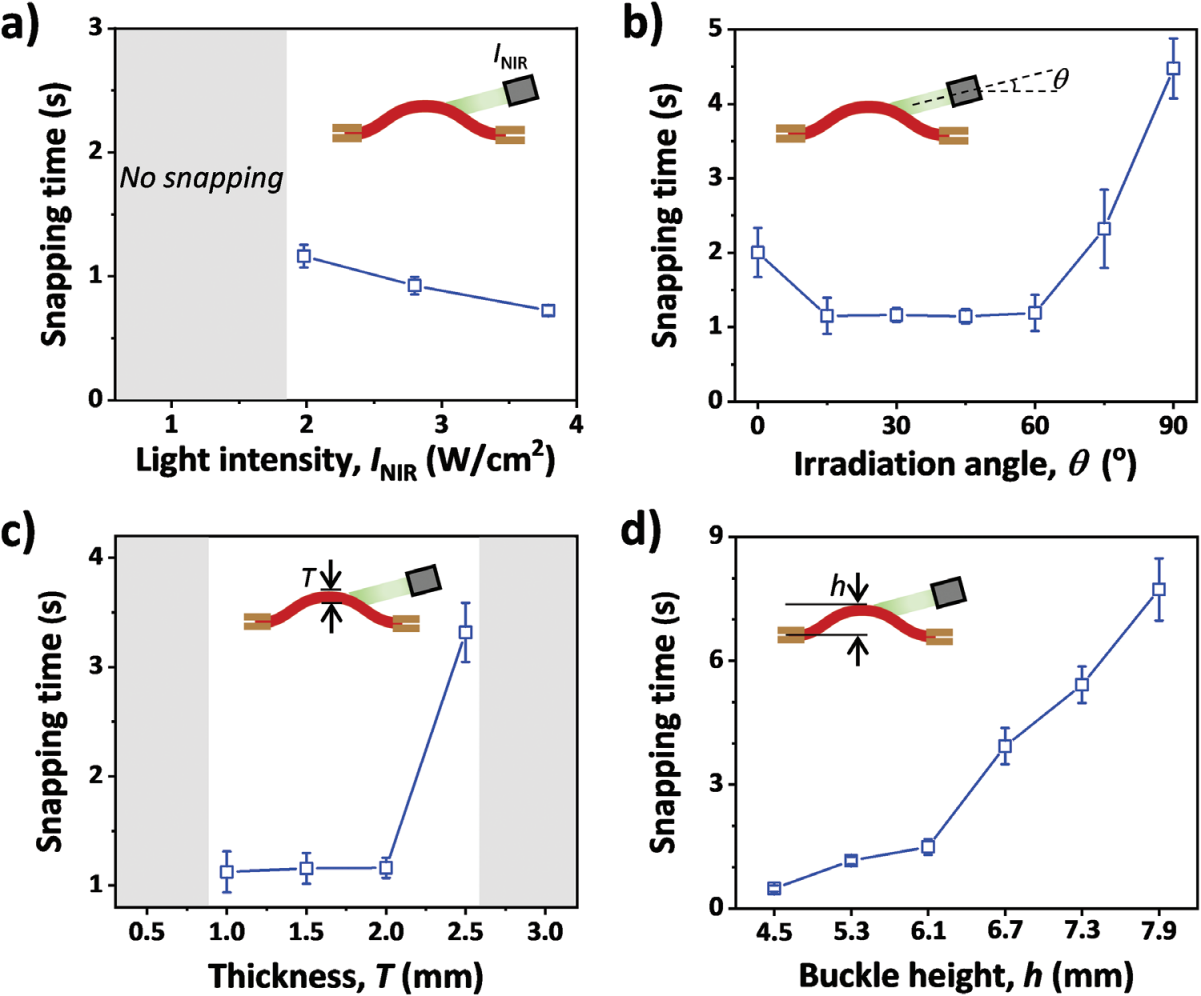
Variations of snapping time as functions of the power intensity (a) and irradiation angle (b) of the laser, as well as the thickness (c) and buckle height (d) of the gel. To examine the effect of each parameter, the others are kept constant with referenced power intensity of 1.98 W cm^−2^, irradiation angle of 30°, gel thickness of 2 mm, and buckle height of 5.3 mm. Gel strips have a constant length of 2 cm. The gray region represents the conditions lacking a snap‐through transition in the gel.

We further investigate the effects of gel properties and irradiation conditions on the snapping behaviors of the constrained gel. These factors, including the power intensity and irradiation angle of the laser, as well as the thickness and buckle height of the gel, exert influences on the snapping through different manners. For example, the power intensity or irradiation angle of the laser influences the local temperature or the irradiation area, respectively, without changing the initial geometry of the buckled gel. Therefore, the energy barrier against the snap‐through transition that is related to gel properties and buckle configuration is almost constant. As the intensity of the laser, *I*_NIR_, increases from 1.98 to 3.79 W cm^−2^, the snapping time, *t*_s_, decreases from 1.16 to 0.7 s (**Figure** **3**a). This is because the gel takes more time to reach the LCST and overcome the energy barrier under a lower power intensity. When *I*_NIR_ is lower than 1.08 W cm^−2^, the snap‐through transition cannot be triggered by light, because the saturated temperature of the gel is still below the LCST (Figure S4a, Supporting Information). As expected, the irradiation angle also influences the snapping time. When the irradiation angle, *θ*, ranges from 15° to 60°, *t*_s_ is almost constant of ≈1.2 s (Figure 3b). However, *t*_s_ increases to ≈2 s, when *θ* is 0° or 75°; it further increases to ≈4.5 s when *θ* = 90°. The increased snapping time of the gel with *θ* = 0° is attributed to the decreased irradiation area of the actuated buckle with a decreasing height. The increased snapping time of the gel with *θ* = 75° or 90° is probably because of the initially slow deformation required to flatten the gel before the snap‐through transition.^[^ ^9^ ^]^

Regarding the gel thickness, it influences the photothermal gradient, the bending stiffness, and thereby the snapping time of the constrained buckle. When the thickness, *T*, is less than 0.5 mm, the snapping does not happen because the light penetration (*I*_NIR_ = 1.98 W cm^−2^; *θ* = 30°) cannot generate an effective photothermal gradient across the thickness of the gel (Figure 3c). When *T* increases from 1 to 2 mm, *t*_s_ only increases slightly from 1.1 to 1.2 s, because of the compromised effects of enhanced photothermal gradient and increased bending stiffness. When *T* increases further to 2.5 mm, *t*_s_ increases to 3.3 s due to the higher energy barrier against the snap‐through transition of the constrained gel. The gel with *T* = 3 mm does not exhibit snapping, because the energy barrier is too high to overcome. The buckle height, *h*, of an identical gel strip, which is related to energy barrier, also influences the snap‐through transition. As shown in Figure 3d, *t*_s_ increases from 0.5 to 7.7 s, when *h* increases from 4.5 to 7.9 mm. In a buckle with a larger height (corresponding to a larger curvature), more elastic energy is required to be accumulated to surpass the barrier for the snap‐through transition of the constrained gel. Therefore, it requires a longer period of irradiation time to trigger the snapping.

### 2.3 Versatility and Synergistic Effect of Light‐Actuated Snapping

**Figure 4:**
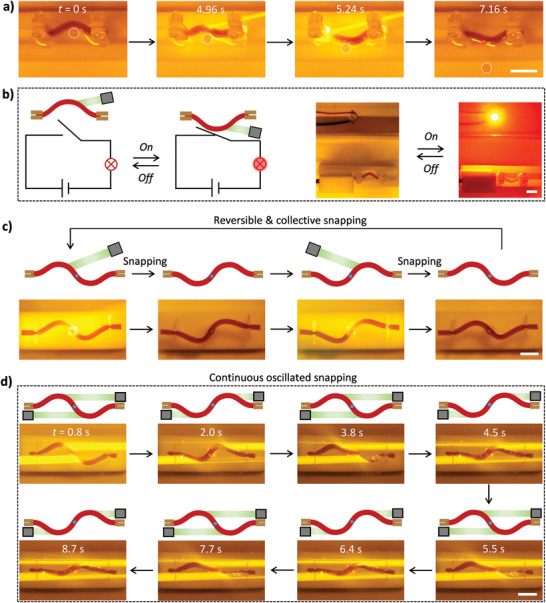
a) Snapshots to show the kicking of a ball by the light‐triggered snapping of the buckled gel. b) Schematic and experimental observations of a device with the buckled gel to control the switch state of the circuit. Length and thickness of the gels in (a) and (b) are 20 and 2 mm, respectively. c) Schematic and Snapshots showing the synergistic snapping of an S‐shaped slender gel. The light‐triggered snap‐through transition of one buckle leads to cooperative snapping of the other buckle. d) Schematic and Snapshots showing the continuous oscillated snapping of the S‐shaped slender gel under static irradiation of two parallel laser beams. Length and thickness of the gels in (c) and (d) are 40 and 2 mm, respectively. Power intensity, 1.98 W cm^−2^. Scale bars, 1 cm.

The light‐actuated snap‐through transition of hydrogels has a set of advantages, such as fast action, large stroke, and no additional energy required to maintain the resultant configuration. These marked features enable the design of versatile soft actuators based on the constrained gel capable of snapping upon remote stimulations. Several proof‐of‐concept experiments are designed to demonstrate the versatility. As shown in **Figure** **4**a and Movie S3 (Supporting Information), the snapping of the gel strip with a large stroke is harnessed to kick a plastic ball. The ball with a diameter of 4 mm is placed at the concave of the buckled gel. After laser irradiation at the other side of the buckle for a few seconds, snap‐through transition is triggered, which generates a large stroke to kick the ball away from the gel with a maximum speed of 28 mm s^−1^. The snapping of the buckled gel can also be used as an optical switch. As shown in Figure 4b and Movie S4 (Supporting Information), the circuit switch is in the open state, when the gel buckles upward. After laser irradiation for a while, snapping of the buckle takes places, accompanied by a quick push on the switch to turn on the light emitting diode. When compared to the switches based on low‐speed deformations of soft actuators,^[^ ^10^ ^]^ the switch based on snap‐through transition is more precise and efficient.

Besides snapping of a single element, multiple units can also be designed in a soft actuator for versatile applications. An interesting phenomenon found in this work is that the snapping of two neighboring buckles with opposite directions in a slender gel shows a synergistic effect. As shown in Figure 4c, the slender gel strip is deformed into an S‐shape, with two ends being clamped and the middle fixed by a fine needle. Therefore, the middle part of the constrained gel can rotate freely rather than have translational motions. Accordingly, deformations of each separate part will influence the other configuration. When the left buckle is irradiated from the top, snapping is triggered with this unit buckling downward. Strikingly, the right buckle spontaneously snaps upward, and the slender gel forms a new configuration of reverse S‐shape. Apparently, this cooperativity is regulated by mechanics, because there is no laser irradiation on the right buckle. If the two units buckle to the same direction, the connection region (i.e., the middle region fixed by the needle) will have a large curvature and thus very high elastic energy.^[^ ^11^ ^]^ In alternative, the right unit buckles to the opposite direction to minimize the total elastic energy of the intact gel. Such cooperative snapping of the two units can also be triggered by laser irradiation on the right buckle (Movie S5, Supporting Information). It can also be repeated for a number of times by switching the buckle unit with imposed laser irradiation.

Self‐sustained and continuous snapping of the S‐shaped gel is also realized by using a pair of parallel‐arranged laser beams. As shown in Figure 4d and Movie S6 (Supporting Information), one laser beam is placed atop the clamps to irradiate the upward buckle, while the other is placed below the clamps to irradiate the downward buckle. The local irradiation results in snap‐through transition of each unit to the opposite direction, which spontaneously exchanges the irradiation condition for each buckle. The irradiation from the opposite side leads to snapping of the buckle to the other direction. Therefore, oscillated snapping takes place in this S‐shaped gel, which lasts for at least 8 min. We should note that degraded performance appears after long‐term laser irradiation, due to the gradual contraction of the gel. Such light‐triggered oscillated snapping is realized in hydrogels for the first time, to our best knowledge, which may provide new avenue for the design of autonomous soft robots.

## 3 Conclusion

In summary, fast and reversible snap‐through transition is realized in a buckled hydrogel upon light irradiation, affording speedy action and large stroke that are highly desired for the design of hydrogel actuators and robots. The anisotropic nanocomposite gel is facilely prepared by flow‐induced orientation of the NSs followed by photopolymerization to fix the oriented structure, which exhibits fast responses to heating and light. The bistable structure of the slender gel is developed by clamping the two ends at a distance appropriate to form a buckle. Upon light irradiation, the gel buckle intends to bend to the opposite direction. Yet a spontaneous movement is hampered by the geometric constrain. After the accumulated elastic energy is above a threshold, however, a snap‐through transition takes place. The snapping is reversible and highly modulable by controlling the gel geometry and irradiation conditions. Energy release of the gel in a short time can be used to execute tasks, such as kicking a ball and remotely controlling the switch state. Besides, collective snapping of a slender gel with two opposite buckles is also realized by laser irradiation on one buckle due to the mechanical coupling of neighboring units. The gel with two opposite buckles even shows oscillated snapping under horizontal irradiation of two parallel laser beams. The collective and synergetic behaviors of shape morphing should merit developing advanced soft machines. The light‐manipulated robust snap‐through transition of hydrogels should be informative for the designs of metamaterials, autonomous soft robots, and energy harvests for specific applications.

## 4 Experimental Section

### Materials

*N*‐isopropylacrylamide (NIPAm) was used as received from Tokyo Chemical Industry Co., Ltd. *N*,*N*′‐methylenebis(acrylamide) (MBAA) was purchased from Aladdin Chemistry Co., Ltd. Lithium phenyl‐2,4,6‐trimethylbenzoylphosphinate (LAP) was synthesized according to the reported protocol.^[^ ^12^ ^]^ Fluorohectorite [Na_0.5_][Li_0.5_Mg_2.5_][Si_4_]O_10_F_2_ nanosheets (NSs) were synthesized by adapting the reported procedure.^[^ ^13^ ^]^ The NS with thickness of ≈1 nm and size of ≈20 µm had a large aspect ratio of ≈20 000, as characterized by atomic force microscopy (Figure S9, Supporting Information). The aqueous suspensions of NSs were prepared by adding prescribed amount of NS powders to water followed by oscillating at room temperature for 15 days. The high charge density (≈1.1 nm^−2^) of NSs enabled repulsive osmotic delamination of the powers into single lamellae in water. Gold nanoparticles (AuNPs) were synthesized according to the reported process,^[^ ^14^ ^]^ and had a diameter of 13.4 ± 1.2 nm, as measured from the transmission electron microscope image (Figure S10, Supporting Information). Millipore deionized water was used in all the experiments.

### Synthesis of Anisotropic Gels

The precursor suspension was prepared by dissolving a prescribed amounts of NIPAm (1 mol L^−1^), MBAA (2 mol%, relative to NIPAm), LAP (0.3 mol%, relative to NIPAm), and AuNPs (0.01–0.05 wt.%) in the homogeneous aqueous suspension of NSs with the content of 1 wt.%. The precursor suspension was injected into a glass tube with rectangular cross‐section (width of 4 mm; height of 0.5–3 mm) at a flow rate of 3 mm s^−1^, resulting in orientation of the NSs along the tube. The flow‐induced orientation of NSs in the precursor could maintain for at least 20 min (Figure S11, Supporting Information), which was immobilized by photopolymerization after immediate exposure to UV light for 30 s. Thus obtained anisotropic hydrogels were incubated into large amount of water for several days to remove the residuals and achieve the equilibrium state at room temperature.

### Characterizations

Absorption spectra of the AuNP suspension and the nanocomposite gel were obtained by using a UV‐1800 spectrometer (Shimadzu Corp., Japan). The AuNP suspension and the anisotropic hydrogel were kept in a quartz cuvette with an optical path of 1 mm for the measurements at room temperature. To monitor the photothermal effect of the nanocomposite gel, the local temperature of the gel under the irradiation of green light (520 nm) was measured by an infrared imager (Fotric 285). A movie is recorded during the laser irradiation, and the varying temperature with the time is analyzed from the movie. Ordered structures of the nanocomposite gels were observed under a polarizing optical microscope (POM; LV100N POL, Nikon) with and without a 530 nm tint plate. The gel was cut into strips with a thickness of ≈2 mm for observations from different directions. SAXS measurements of the anisotropic gel were conducted on the BL16B beamline with an X‐ray wavelength of 0.124 nm at Shanghai Synchrotron Radiation Facility. The size of the beam spot was 172 × 172 µm^2^, and the sample‐to‐detector distance was 2188 mm. Intensity‐azimuthal plot of the gel was used to calculate the orientation degree (*π*) of the NSs within the gel according to the equation of *π* = (180 − *H*)/180, where *H* is the halfwidth of the scattering peak in the intensity‐azimuthal plot from the selected equatorial reflection.^[^ ^15^ ^]^

Anisotropic response of the gel to heating was characterized by measuring the variations of gel's dimensions. The gel was transferred from a 25 °C water bath to a 40 °C water bath, and the variations of gel's dimensions were recorded by a movie. The variations in length along the *x*‐direction and *y*‐direction of the gel are calculated as *S*_x_ = *X*_1_/*X*_0_ and *S*_y_ = *Y*_1_/*Y*_0_, respectively, in which *X* and *Y* are the lengths of the gel in *x* and *y* directions. The subscript numbers 1 and 0 correspond to the deformed and original states, respectively, of the gel.

External force–induced snap‐through transition of the constrained hydrogel strip was investigated by using a tensile tester to generate controllable compressive strain on the buckle at room temperature. The gel strip was clamped at two ends to form a buckled structure. A cylinder glass with the diameter of 6 mm was fixed on the tester as the indenter, which moved downward to compress the gel buckle with a constant rate of 5 mm min^−1^. After certain compressive strain, the buckled gel snapped downward to the other side. The force–displacement curve was recorded. Similar snap‐through transition was triggered by light irradiation on the constrained gel strip with certain irradiation angle and power intensity. The snapping process of the constrained gel strip was recorded by a digital camera. The variation of the buckle height during the snap‐through transition was then analyzed from the snapshots of the movie. Local temperature of the gel under light irradiation was measured by an infrared imager.

## Conflict of Interest

The authors declare no conflict of interest.

## Supporting information

Supporting Information

Supplemental Movie 1

Supplemental Movie 2

Supplemental Movie 3

Supplemental Movie 4

Supplemental Movie 5

Supplemental Movie 6

## Data Availability

The data that support the findings of this study are available from the corresponding author upon reasonable request.
