# Supplementary material for: Reversible Snapping of Constrained Anisotropic Hydrogels Upon Light Stimulations
Source: Adv Sci (Weinh). 2024 May 5;11(26):2402824. doi: 10.1002/advs.202402824 (PMC11234394; doi:10.1002/advs.202402824)
Supplement: Supplementary file 1 — Supporting Information [file ADVS-11-2402824-s006.pdf]

ADVANCED SCIENCE
Open Access

Supporting Information

for *Adv. Sci.*, DOI 10.1002/advs.202402824

Reversible Snapping of Constrained Anisotropic Hydrogels Upon Light Stimulations

Chen Fei Dai, Qing Li Zhu, Olena Khoruzhenko, Michael Thelen, Huiying Bai, Josef Breu, Miao Du, Qiang Zheng and Zi Liang Wu**

Supporting Information

Reversible Snapping of Constrained Anisotropic Hydrogels upon Light Stimulations

Chen Fei Dai, Qing Li Zhu, Olena Khoruzhenko, Florian Puchtler, Huiying Bai, Josef Breu, Miao Du, Qiang Zheng, Zi Liang Wu**

C. F. Dai, Q. L. Zhu, H. Bai, M. Du, Q. Zheng, Z. L. Wu

Ministry of Education Key Laboratory of Macromolecular Synthesis and Functionalization

Department of Polymer Science and Engineering

Zhejiang University

Hangzhou 310058, China

E-mail: wuziliang@zju.edu.cn

O. Khoruzhenko, F. Puchtler, J. Breu

Bavarian Polymer Institute and Department of Chemistry

University of Bayreuth

Universitätsstrasse 30, Bayreuth 95440, Germany

E-mail: Josef.Breu@uni-bayreuth.de

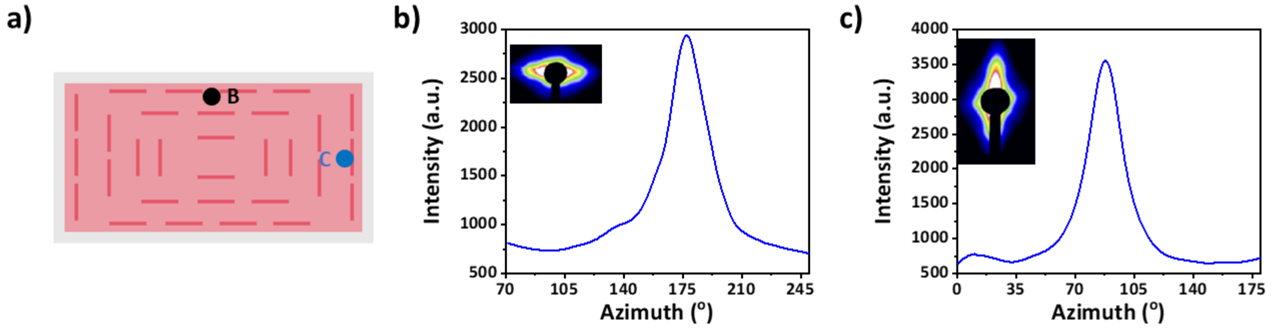

Figure S1. (a) Schematic to show the directed positions in the small-angle X-ray scattering (SAXS) measurements of the anisotropic gel. (b,c) Scattering intensity-azimuth plots of the gel in position B (b) and position C (c) schemed in (a). The insets in (b) and (c) are the 2D SAXS patterns. The orientation degrees of the NSs in the position B and position C of the gel are 0.79 and 0.84, respectively.

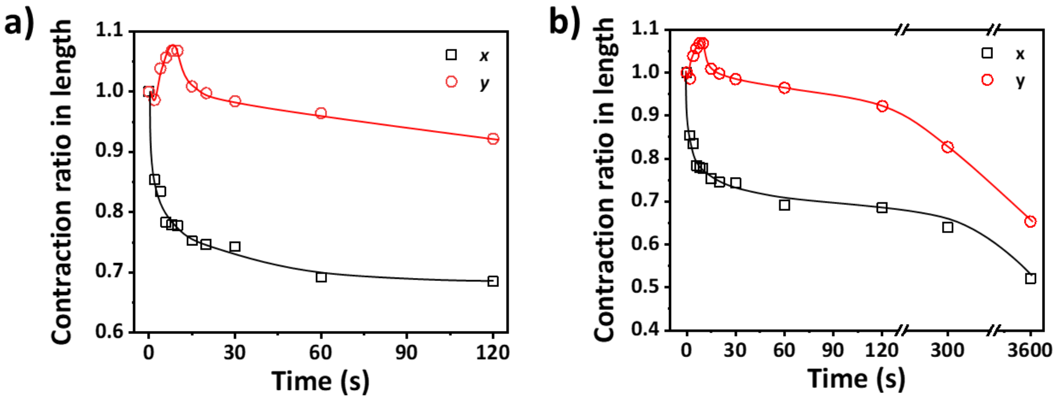

Figure S2. Varying dimensions of the anisotropic hydrogel along *x*-direction and *y*-direction after being transferred from 25 to 40 °C water bath for 2 min (a) and 60 min (b).

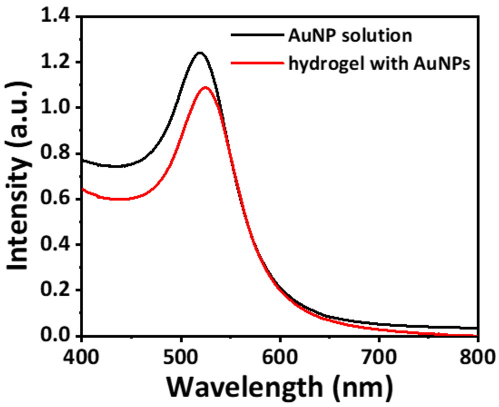

Figure S3. Absorption spectra of the aqueous suspension of 0.03 wt% AuNPs and the nanocomposite hydrogel containing 1 wt% NSs and 0.03 wt% AuNPs at room temperature.

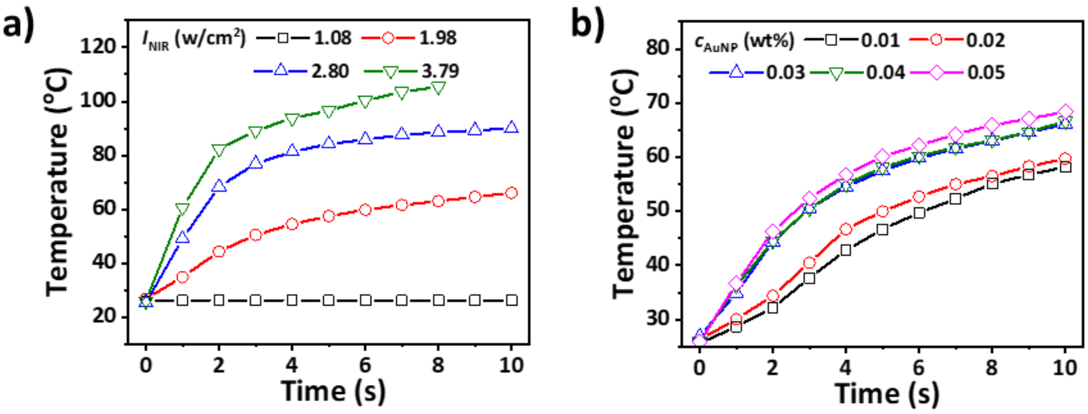

Figure S4. Varying local temperature of the nanocomposite gels under the irradiation of 520 nm green laser with different power intensity I_{NIR} (a) and different AuNP content c_{AuNP} (b). Content of NSs in the gels is kept as 1 wt%, while c_{AuNP} in (a) is 0.03 wt% and I_{NIR} in (b) is 1.98 W/cm^2.

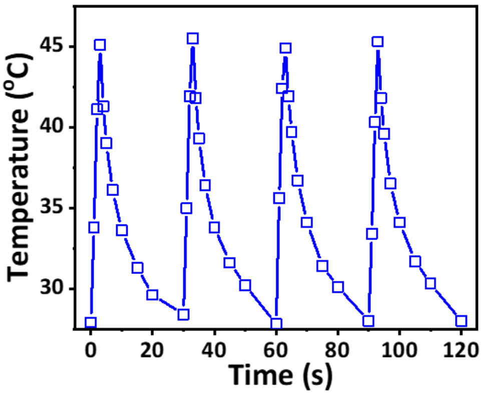

Figure S5. Varying local temperature of the gel under cyclic irradiation of 520 nm laser with power intensity of 1.98 W/cm^2. Contents of NSs and AuNPs in the gel are 1 wt% and 0.03 wt%, respectively.

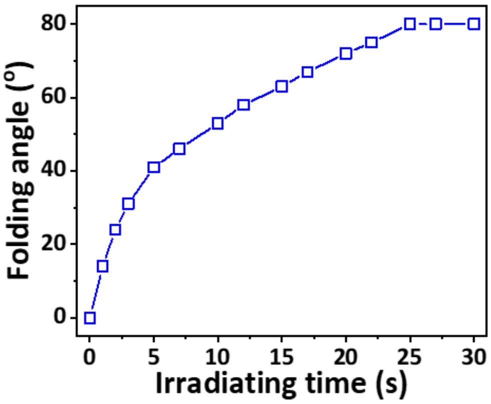

Figure S6. Varying folding angle of the gel strip under the irradiation of 520 nm green laser with power intensity of 1.98 W/cm^2. Contents of NSs and AuNPs in the gel are 1 wt% and 0.03 wt%, respectively.

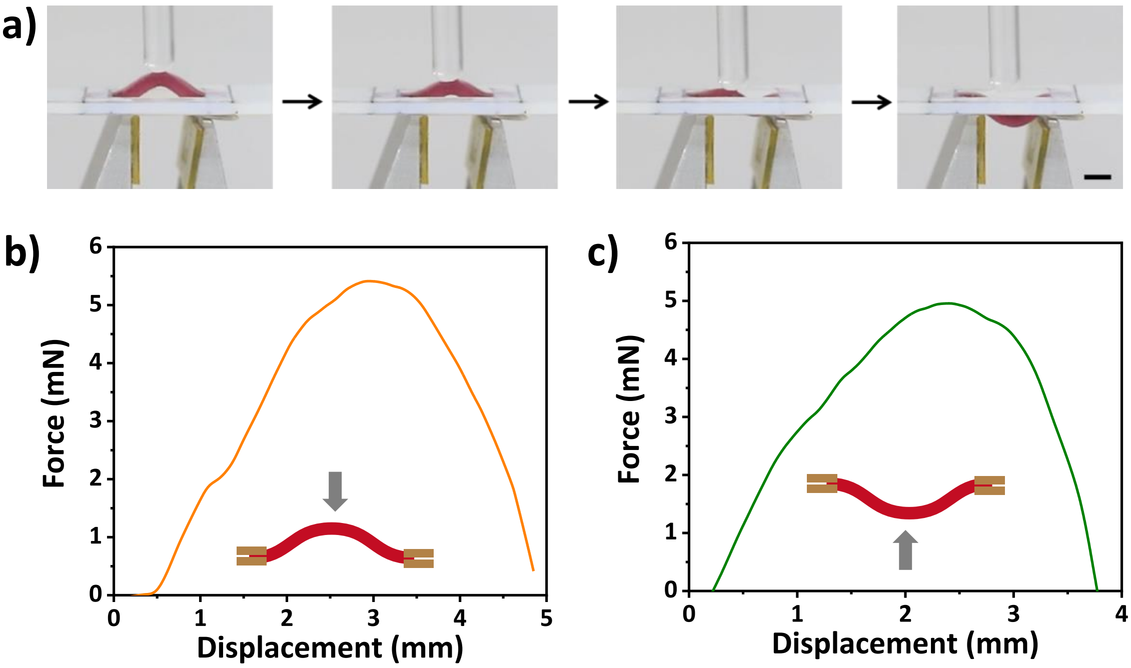

Figure S7. Reversible snapping of buckled hydrogel induced by external force. (a) Snapshots to show the deformation and snapping transition of the buckled hydrogel compressed by an indenter. The gel experienced an asymmetrical deformation before the occurrence of snapping transition. Scale bar: 5 mm. (b) Force-displacement curves of reversible snapping of hydrogel induced by external force. The hydrogel was flipped to make the buckle face upward against the indenter for the next-round force-induced snapping transition.

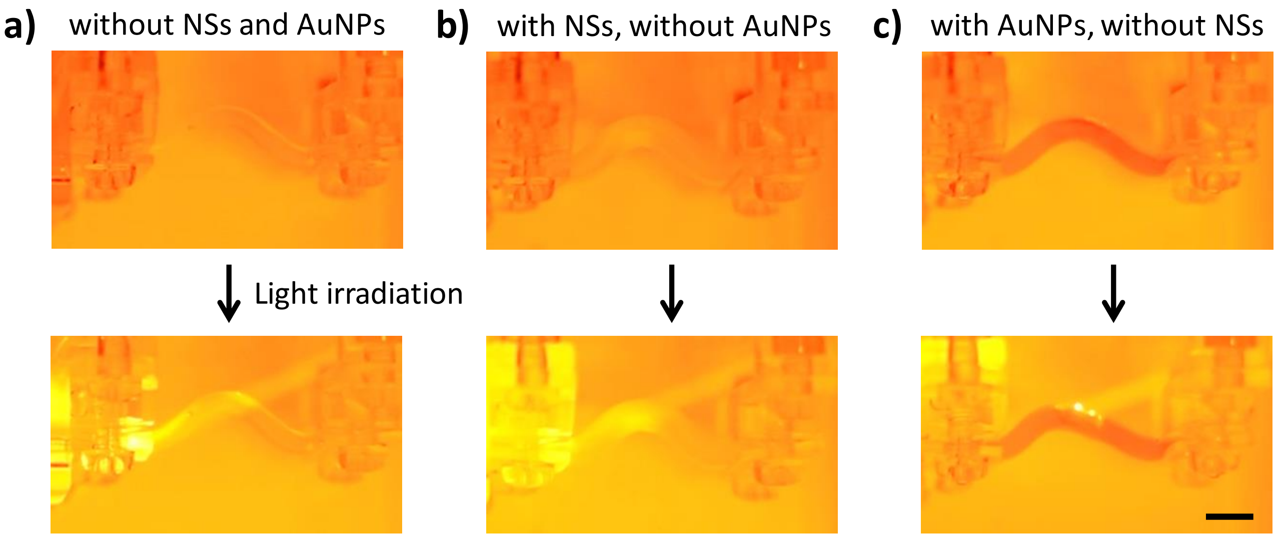

Figure S8. Digital photos to show the configurations/deformations of the constrained gel strips without NSs and AuNPs (a), with NSs but without AuNPs (b), and with AuNPs but without NSs (c) under laser irradiation. The upper and bottom photos show the state of the gels before and after the laser irradiation for 60 s. The results indicate that no snapping occurs in these gels. Scale bar, 5 mm.

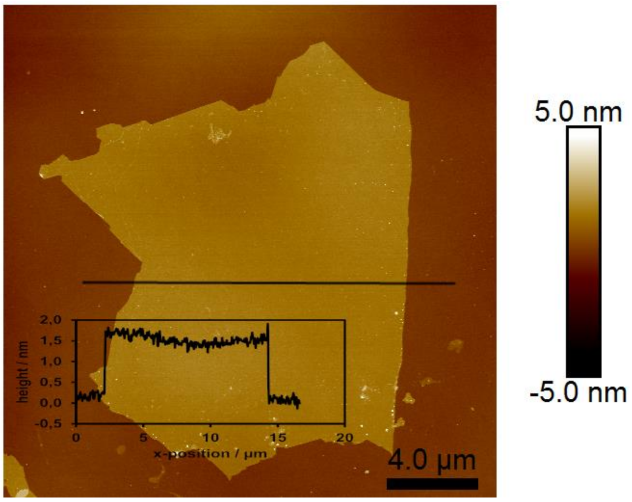

Figure S9. Atomic force microscope (AFM) image of the NS. Inset shows the height profile along the black line.

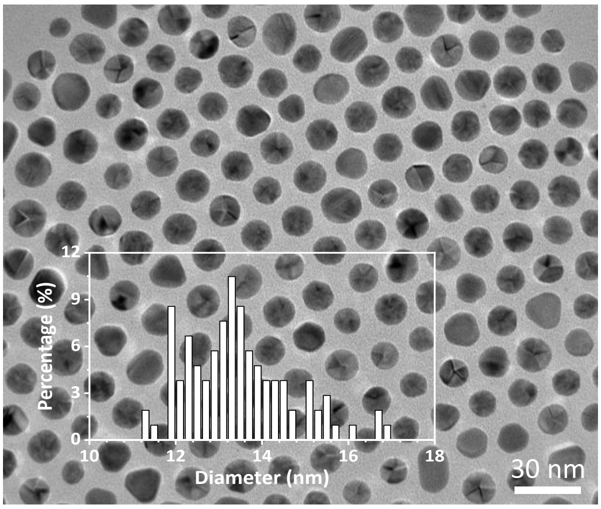

Figure S10. Transmission electron microscope image of the AuNPs. Inset shows the statistical diameter of the AuNPs (n = 100). The diameter of AuNPs is 13.4 ± 1.2 nm (mean ± standard deviation).

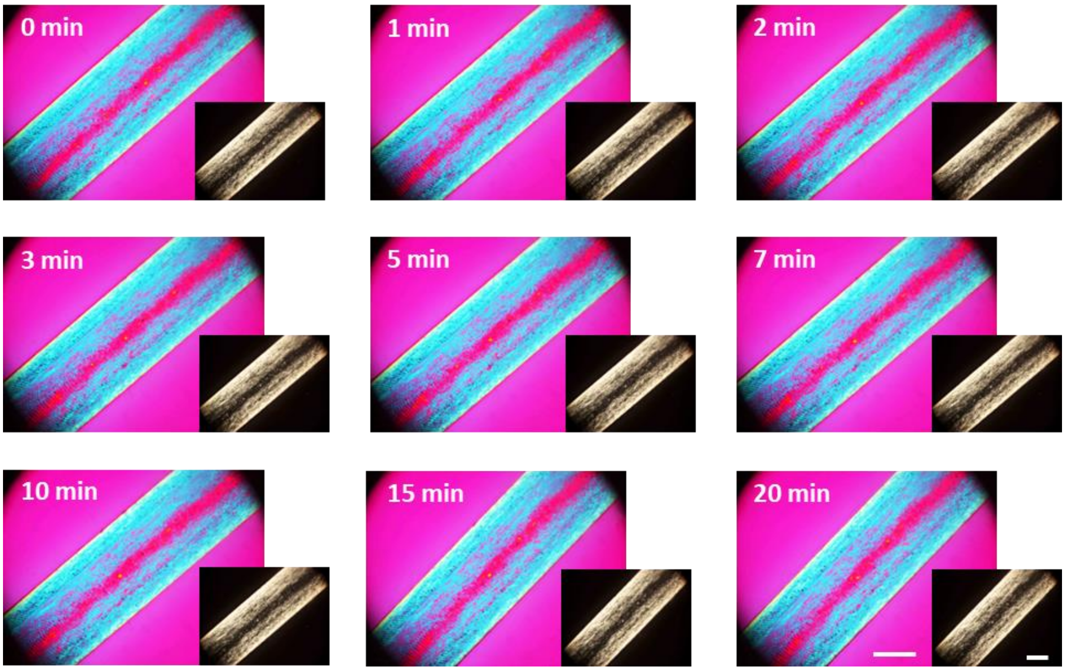

Figure S11. Structural relaxation of the ordered structure after flow-induced alignments of NSs along the tube. The NSs with content of 1 wt% in the precursor suspension are oriented after injection with a flow rate of 3 mm/s at room temperature, and the structural relaxation process is observed under polarizing optical microscope. The relaxation time is noted in images. Scale bar, 2 mm.

Table S1. The distance between the clamped ends and corresponding buckle height of the gel strip with an identical length of 20 mm in Figure 3d.

Height (mm)	Distance (mm)
4.5	19
5.3	18
6.1	17
6.7	16
7.3	15
7.9	14

Movie S1. Reversible folding of the anisotropic hydrogel strip upon cyclic light irradiation.

Movie S2. Reversible snapping of the buckled hydrogel strip upon cyclic light irradiation.

Movie S3. Light-actuated snapping of the buckled hydrogel to kick a ball.

Movie S4. Light-actuated snapping of the buckled hydrogel to control the state of the circuit.

Movie S5. Synergistic snapping of the S-shaped hydrogel upon light irradiation.

Movie S6. Continuous oscillated snapping of the S-shaped hydrogel upon light irradiation of two parallel laser beams.
